# Inter-group alliance dynamics in Indo-Pacific bottlenose dolphins (*Tursiops aduncus*)

**DOI:** 10.1007/s10071-023-01804-y

**Published:** 2023-06-30

**Authors:** Whitney R. Friedman, Michael Krützen, Stephanie L. King, Simon J. Allen, Livia Gerber, Samuel Wittwer, Richard C. Connor

**Affiliations:** 1 Department of Environmental Studies, University of California Santa Cruz, Santa Cruz, 95064 USA; 2 Department of Cognitive Science, University of California San Diego, San Diego, 92093 USA; 3 Evolutionary Genetics Group, Department of Evolutionary Anthropology, University of Zurich, Zurich, Switzerland; 4 School of Biological Sciences, University of Bristol, Bristol, BS8 1TQ UK; 5 School of Biological Sciences, University of Western Australia, Crawley, WA 6009 Australia; 6 Australian National Wildlife Collection, National Research Collections Australia, CSIRO, Canberra, ACT 2601 Australia; 7 Biology Department, University of Massachusetts Dartmouth, North Dartmouth, MA 02747 USA; 8 Institute of Environment and Department of Biological Sciences, Florida International University, North Miami, FL 33181 USA

**Keywords:** Affiliative behavior, Coalitions, Differentiated relationships, Social complexity, Strategic alliances, Bottlenose dolphins

## Abstract

The social intelligence hypothesis holds that complex social relationships are the major selective force underlying the evolution of large brain size and intelligence. Complex social relationships are exemplified by coalitions and alliances that are mediated by affiliative behavior, resulting in differentiated but shifting relationships. Male Indo-Pacific bottlenose dolphins in Shark Bay, Australia, form three alliance levels or ‘orders’, primarily among non-relatives. Strategic alliance formation has been documented *within* both first- and second-order alliances and *between* second-order alliances (‘third-order alliances’), revealing that the formation of strategic inter-group alliances is not limited to humans. Here we conducted a fine-scale study on 22 adult males over a 6-year period to determine if third-order alliance relationships are differentiated, and mediated by affiliative interactions. We found third-order alliance relationships were strongly differentiated, with key individuals playing a disproportionate role in maintaining alliances. Nonetheless, affiliative interactions occurred broadly between third-order allies, indicating males maintain bonds with third-order allies of varying strength. We also documented a shift in relationships and formation of a new third-order alliance. These findings further our understanding of dolphin alliance dynamics and provide evidence that strategic alliance formation is found in all three alliance levels, a phenomenon with no peer among non-human animals.

**Supplementary Information:**

The online version contains supplementary material available at 10.1007/s10071-023-01804-y.

## Introduction

The social intelligence hypothesis (SIH) posits that the complexities of social relationships provided the selection pressure for the evolution of intelligence and large brain size (Jolly 1966; Humphrey 1976; Alexander 1979, 1989; Byrne and Whiten 1988; Dunbar 1992, 1998). Alliances and coalitions, defined as two or more individuals cooperating against conspecifics, repeatedly so in the case of alliances, are often singled out as exemplifying complex relationships (e.g.Chapais 1995; Connor 2007; Harcourt and de Waal 1992). Alliances demonstrate relational complexity to the extent that they are *strategic*, meaning that individuals have options to choose or compete for different possible allies and that those choices carry risk (Connor 2007; Lukas and Clutton-Brock 2018). If potential alliance partners are equivalent, then there is little risk in making the wrong choice, but increasing the number of options, and variance in the fitness consequences of those options, should increase selection for social intelligence (Connor 2007).

The importance of options in alliance formation can be illustrated with the two alliance levels found in many primate species: those formed within social groups and those against other groups. The greatest strategic challenges are found within groups, where individuals may have multiple options, and rivals compete for the same allies using a combination of aggression against rivals and affiliative interactions with preferred partners (e.g. Bray and Gilby 2020; de Waal 1982; Silk et al. 2004). Especially among non-kin primates, individuals are free to shift allies, demonstrating what Nishida (1983) called ‘allegiance fickleness’. These strategic options are reduced when alliances are primarily based on kin, a phenomenon typical of cercopithecine primates (Connor 2007; Cords 2012; Lukas and Clutton-Brock 2018).

Under the SIH, selection should favor cognitive abilities to assess different options, such as recognition of third-party relationships (Harcourt and de Waal 1992) and, at least in the case of humans, social scenario building (Alexander 1989). Risk is enhanced when there is uncertainty in the status of third-party relationships (Connor 2007; Aureli et al. 2008; Ramos-Fernandez et al. 2018). For example, the decision to attack an opponent defeated previously may be a mistake if, in the interim, that individual has developed new, powerful allies.

Inter-group interactions are generally hostile (but see Furuichi 2020) and may be risky, e.g., chimpanzee killing (Wrangham et al. 2006). Further, more dangerous inter-group conflicts may increase mutual dependence among group members and increase the fitness consequences of within-group social decisions (Alexander 1979; Connor 2007). However, the options available in inter-group encounters are typically limited to the decision to participate or not (e.g., lion ‘laggards’, Heinsohn and Packer 1995; Willems et al. 2013). Chimpanzees are more likely to join an inter-group conflict when the number of participants is larger and includes maternal kin or social partners (Samuni et al. 2022). This indicates a greater willingness to take risks to support key within-group allies. Inter-group interactions in primates might approach the complexity of within-group interactions if they included more options, such as forming alliance bonds with either of two neighboring groups for the purpose of attacking the other. However, such strategic ‘alliances of alliances’ have been found only in humans (Alexander 1989; Chapais 2013; Macfarlan et al. 2014) and a population of Indo-Pacific bottlenose dolphins (*Tursiops aduncus*) in Western Australia (Connor et al. 1992a, 2011, 2022; Connor 2007).

The male bottlenose dolphins in Shark Bay participate in up to three levels or *orders* of alliances in a large, open society of resident dolphins with a dynamic fission–fusion grouping pattern (Connor and Krützen 2015; Gerber et al. 2020, 2021; King et al. 2021). Males have larger ranges than females but retain their natal range in their adult home range (Connor et al. 2000; Krützen et al. 2004; Tsai and Mann 2013) and both sexes exhibit a continuous mosaic of overlapping home ranges (Watson-Capps 2005; Randić et al. 2012; O’Brien et al. 2020). *Second-order alliances* contain 4–14 males, may endure for decades, and are considered the core unit of male social organization in Shark Bay. *First-order alliances* consist of pairs or trios of adult males from within the second-order alliance. *Third-order alliances* are comprised of two second-order alliances (Connor and Krützen 2015).

Alliances are defined both by quantitative measures of association, as well as functional behaviors. Males in all three levels of alliance associate preferentially as demonstrated by cluster analysis and permutation tests (Connor et al. 1992a, 2011; King et al. 2021). The functions of the three levels of male alliance formation in Shark Bay are well established. Males in first-order alliances work together to sequester and consort single estrus females (Connor et al. 1992a, b). Second- and third-order alliances are functionally identical; cooperation to attack and defend against other alliances in contests over female consorts (Connor et al. 1992a, b, 2011). The strength of a male’s connection with his third-order allies significantly influences the maximum duration of his consortships; where third-order allies provide important ‘backup’ given that second-order alliances vary in size and members are often dispersed over a wide area (Connor et al. 2022).

The distinction between second- and third-order alliances is based on cluster analysis of association data, the nearly exclusive use of second-order allies to form first-order alliances, the exclusive and stable membership of second-order alliances, the uniformly stronger response of males to signature whistle (i.e., identity signal) playbacks of second- vs third-order allies (independently of dyadic bond strength), and the higher rate of signature whistle exchanges between second-order allies (King et al. 2021; Chereskin et al. 2022; Connor et al. 2022). Finally, males are rarely observed with third-order allies without the presence of second-order allies (Connor et al. 2022).

Several markers of strategic within-group coalition and alliance formation typical of primates are present in first- and second-order dolphin alliance interactions. Males in first- and second-order alliances use affiliative interactions to mediate alliance bonds, which develop among mostly unrelated adolescent males of similar age (Connor et al. 2006; Gerber et al. 2020, 2021; Chereskin et al. 2022). Relationships between first- and second-order alliance partners are strongly differentiated but may shift. For example, males often change first-order alliance partners, and rare but consequential changes occur in second-order alliance membership (Connor et al. 1992b, 2001; Connor and Mann 2006; Connor and Krützen 2015). Competition among allies is implied by variation in consortship and paternity success within second-order alliances; and has also been observed in situ within and between first-order alliances (Connor et al. 1992a; Connor and Mann 2006; Connor and Krützen 2015; Gerber et al. 2022).

The finding that allied male dolphins form strategic inter-group alliances (i.e., third-order alliances), similar to humans (Connor et al. 2022), raises intriguing questions about how these between-group cooperative relationships are formed and maintained. Here, we investigated how two second-order alliances maintained their third-order alliance through association and the use of affiliative interactions. Further, in a remarkable case of serendipity during the study, one of these second-order alliances began a new association with another second-order alliance, allowing us to document the formation of a new third-order alliance.

As with first- and second- order alliances, we hypothesized that third-order alliances are maintained by affiliative interactions between individuals belonging to different second-order alliances. If such affiliative interactions occur, how are they distributed? While differentiated relationships are considered a hallmark of social relationship complexity (Freeberg et al. 2012; Bergman and Beehner 2015; Fischer et al. 2017), less relational complexity would be indicated if third-order alliances were, for example, maintained by just 2–3 males in each second-order alliance. If so, affiliative interactions should be restricted to that subset of males. Alternatively, widespread affiliative contact between members of both second-order alliances would suggest that males maintain bonds with all third-order allies even though bond strengths can vary (King et al. 2021); an indicator of greater relational complexity. Dynamic shifts in alliance relationships further evince the complex social landscape formed and maintained by the Shark Bay dolphins, and illuminate the kinds of cognitive demands imposed on individuals operating within this social system.

## Methods

### Location and subjects

Data for this study were collected during 2009–2014 in the eastern gulf of Shark Bay, Western Australia, where our research on Indo-Pacific bottlenose dolphins has been ongoing since 1982. We examined associations and behavioral interactions among 24 adult males (aged 19–37 when the study commenced; Supplementary Table S1) from three frequently sighted second-order alliances (KS, PD, and RR), whose members matured and began consorting females between 1995 and 2004 (Connor and Mann 2006; King et al. 2021). By 2009, the KS alliance had dropped from a peak of 14 to 12 members, the RR alliance from 7 to 5 members, while the PD alliance had remained at 7 members since their formation in the mid-1990s (Connor and Mann 2006; King et al. 2021). Members of these alliances associated as juveniles (Gerber et al. 2020). The third-order alliance between KS and PD, based on preferential association and cooperation in conflict against other alliances, has endured for over 20 years, from at least 2001 when KS members matured and began consorting females (Connor et al. 2011; King et al. 2021).

### Data collection

For association analyses, we used data from group surveys taken when dolphins are sighted. A survey is a minimum 5-min snapshot of dolphin group composition (defined by the 10 m chain rule; Smolker et al. 1992) and predominant group activity. Only association data recorded in the first 5 mins of a survey were used to ensure association measures were comparable across surveys. Individuals were identified based on distinctive dorsal fin characteristics, and all in situ identifications made by trained observers were later confirmed via photo-identification (Würsig and Würsig 1977; Smolker et al. 1992). To avoid cases where males were together because they were attracted to the same food sources, we only used surveys where the predominant group activity was resting, travelling, or socializing. Same day resights (ESM) of groups were excluded, as were sightings of large inter-group conflicts. We restricted all surveys to the period of August–December, aligning with overlaps in mating season and field seasons. Surveys were considered ‘third-order associations’ if they contained at least one individual from two different second-order alliances known to associate as a third-order alliance.

During 2013–2014, we combined aerial and side-video during focal group follows to detect affiliative interactions involving petting, rubbing, and synchrony. Aerial video was recorded using a 2.3 m diameter Allsopp Helikite with a GoPro Hero3 HD video camera (see ESM). Boat-based side-video was recorded using a Canon Vixia HF11 camcorder.

### Associations and confirmation of alliance membership

Associations among individuals were estimated by calculating pairwise half-weight indices (HWI) (Cairns and Schwager 1987; Whitehead 2008) to maintain comparability with prior studies. Alliance membership was confirmed using the average-linkage agglomerative method of cluster analysis using the software programs SOCPROG (Whitehead 2009) and UCINET (Borgatti et al. 2002). This method imposes a hierarchical model onto a social group, and the appropriateness of this model can be tested by calculating a cophenetic clustering coefficient (CCC), which is the correlation between the dyadic association indices and the level at which the dyads are joined on the dendrogram. A CCC greater than 0.8 indicates that the hierarchical model provides a good representation of the social network (Whitehead 2008). Significant groupings within the hierarchical model are detected by calculating a modularity score (Q) for each partition of the network.

### Bond strength within- and between- second-order alliances

To determine how well-connected individual males were within and between their alliances, we calculated the individual level social network metric ‘strength’ (also known as ‘weighted degree’) using half-weight indices. We calculated within- and between-alliance strength values, *wStrength* and *bStrength* respectively, for all individuals. Due to the influence of network size on these two measures, we scaled the strength vales between 0 and 1 by dividing each individual’s within- or between-strength by the maximum strength value in that network, e.g., the maximum strength value in their second-order alliance. Normalized within-alliance strength (*wStrength_N*), therefore, represents how well-connected males are within their second-order alliance, and normalized between-alliance strength (*bStrength_N*) represents how well-connected males are to third-order allies compared to other members of their second-order alliance.

### Temporal changes in bond strength

During the 6-year study, four of the focal males who had been regularly sighted for 10–15 years disappeared and are presumed to have died. Two males from the KS alliance disappeared between the end of the 2010 field season and the start of the 2011 season. Two males from the PD alliance disappeared between the end of the 2012 field season and the start of the 2013 field season. To examine the resulting dynamics of the alliance network, we used a discrete snapshot approach (de Silva et al. 2011; Pinter-Wollman et al. 2014) of three 2-year intervals during which group compositions were stable: (T1: 2009–2010, T2: 2011–2012, T3: 2013–2014).

### Social interactions during inter-alliance fusions

Inter-alliance fusions occurred when members of different second-order alliances joined each other within 10 m or less. We used overhead and side-video to detect affiliative contact behaviors during group fusions, a common context for affiliative interactions in mammals (Nishida 1970; Aureli and Schaffner 2007; Smith et al. 2010; Poole 2011; Luef and Pika 2017). We captured 10 fusion events between two first-order alliances from different second-order alliances. Videos were reviewed by the first author (W.R.F.) for occurrences of affiliative, aggressive, and socio-sexual interactions between second- and third-order allies (Cohen’s kappa = 0.73 between W.R.F. and three trained coders). Affiliative behaviors recorded between individuals included gentle contact behaviors, such as ‘petting’ (contact between the pectoral fin of one individual and any part of the body of another) and ‘rubbing’ (an individual rubs part of its body against the body of another). We also recorded cases where two individuals were close enough to be touching (<1/3 m) but we could not discern actual contact on the video. ‘Synchs’ were scored when two or three individuals, swimming side-by-side less than 2 m apart and with a stagger no greater than 1 m, surfaced or dived synchronously (Connor et al. 2006; McCue et al. 2020). The few recorded instances of aggressive behavior during these events provide interesting context and are discussed in the ESM. In all 10 observed third-order fusion events, one or both first-order alliances were consorting a female. We therefore also examined video for instances where males from one first-order alliance interacted with the consorted female who arrived with the other first-order alliance.

## Results

From 2009 to 2014, 282 surveys included males from the KS, PD and/or RR alliances in rest, travel, or social contexts. Of these, 39 surveys captured third-order associations among KS, PD, and RR males (KS and PD = 26, KS and RR = 11, PD and RR = 2). The third-order KS–PD alliance was persistent throughout the study period; 12% of all surveys in which any KS or PD males were recorded were third-order associations between the KS and PD males. An additional 14 surveys (not included in our analysis) captured these third-order groups in foraging, unknown, and competitive contexts.

**Fig. 1:**
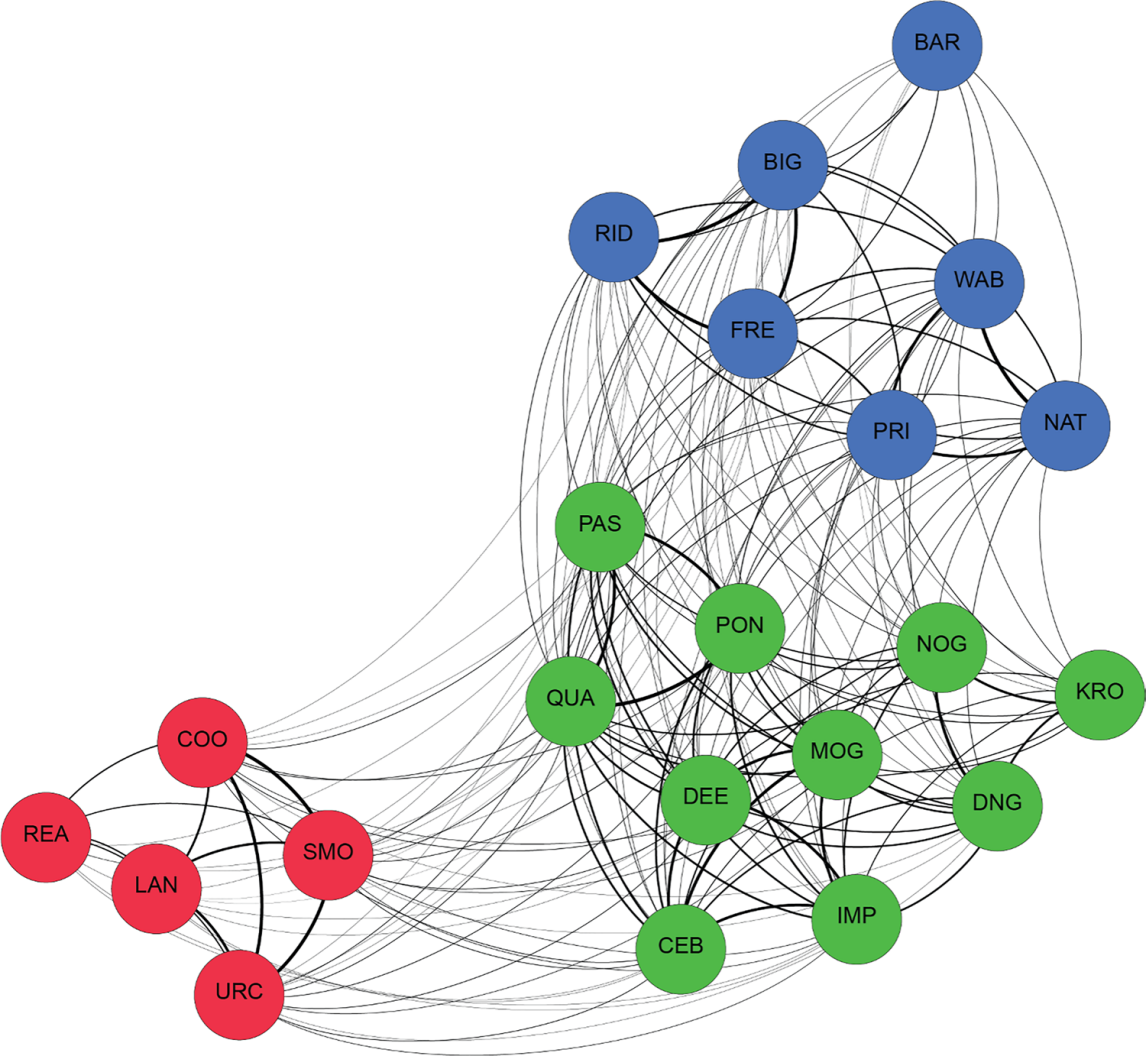
Alliance relationships. Network plot of the three second-order alliances over the entire study period (2009–2014), where males are color-coded by alliance membership (PD = blue, KS = green, RR = red) and thicker edges represent stronger bond strengths (HWI; Supplementary Table S3). Two KS members who disappeared after 2010 are not included (color figure online)

Hierarchical clustering analysis of half-weight association indices (HWI) among the 22 of 24 males present for more than two of the 6 years detected the same three communities (max Q = 0.40 at HWI = 0.20), which had been identified as second-order alliances in the field, i.e., males determined to be in the same second-order alliance through average-linkage hierarchical clustering are the same males we observe consorting females together and defending them from rivals (Connor et al. 2011; Randić et al. 2012; King et al. 2018, 2021; Gerber et al. 2020). The model had a very high cophenetic correlation coefficient (CCC) of 0.98, indicating a good representation of the relationships among these individuals. Alliance membership and network structure is shown in Fig. 1.

### Within and between group strength

**Fig. 2:**
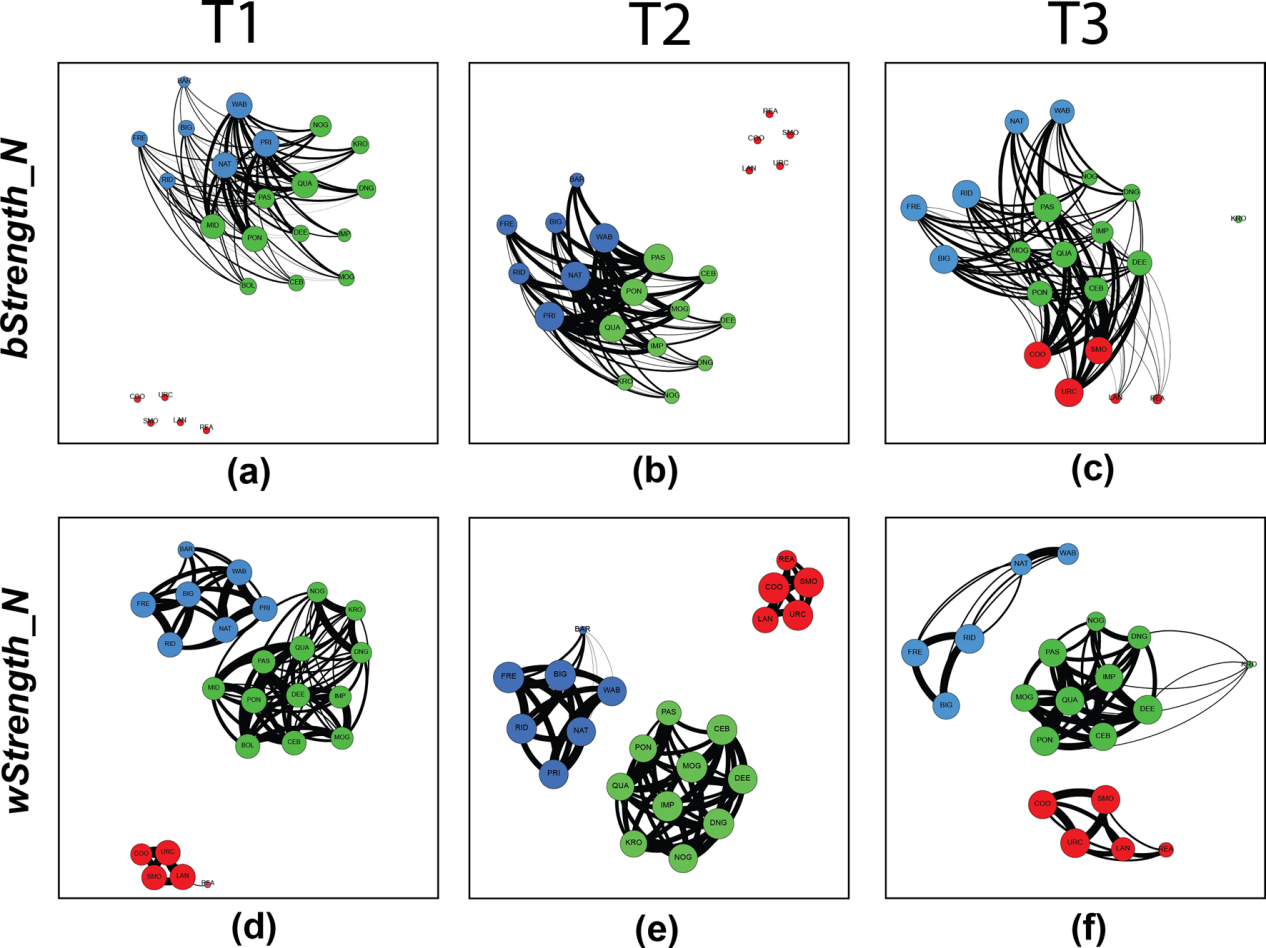
Temporal changes within and between alliance relationships shown by network plots of the three second-order alliances over three 2-year periods: 2009–2010 (T1), 2011–2012 (T2), and 2013–2014 (T3). Males are color-coded by alliance membership (PD = blue, KS = green, RR = red) and thicker lines represent stronger bond strengths (HWI; Supplementary Tables S4–S6). Circle size is based on an individual’s normalized between-group strength value (*bStrength_N*; *panels ***a–c**) or within-group strength value (*wStrength_N*; *panels ***d**–**f**); more ‘central’ individuals are larger and centered within each network (color figure online)

**Fig. 3:**
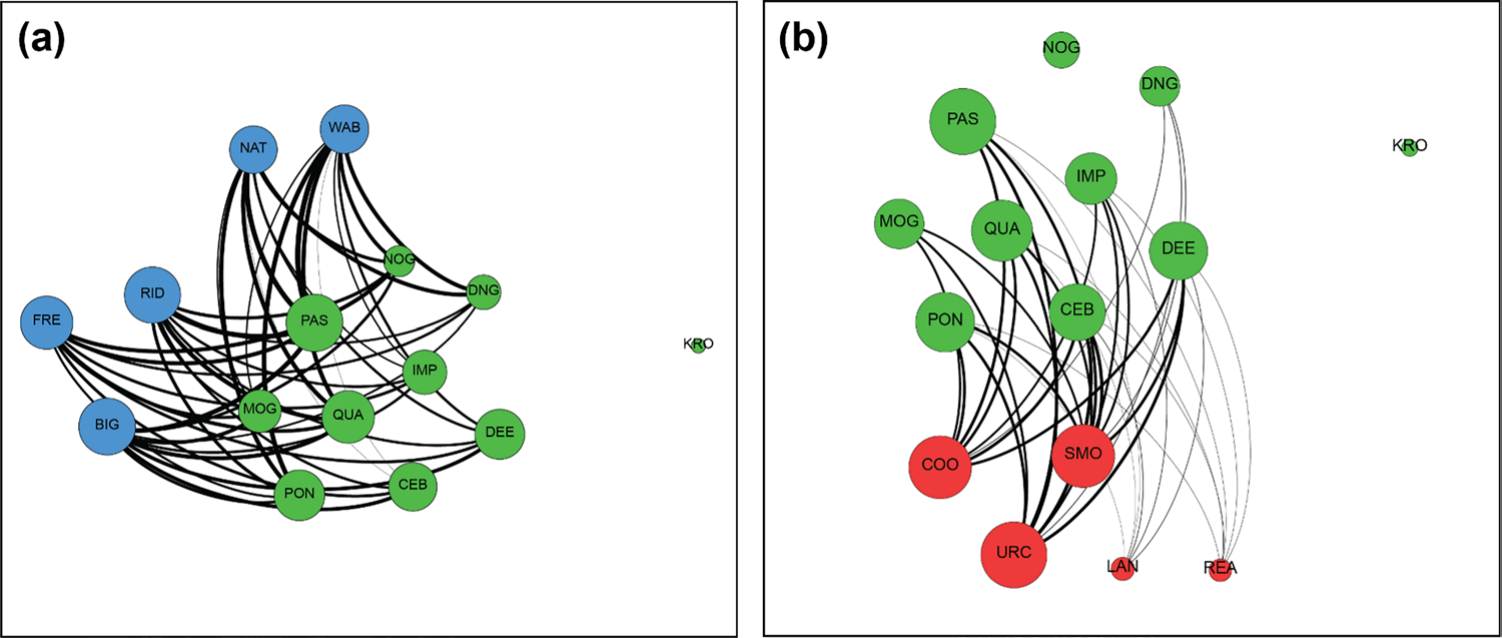
Between-alliance relationships for **a** KS and PD and **b** KS and RR during 2013–2014 (T3). Males are color-coded by alliance membership (PD = blue, KS = green, RR = red), where thicker lines represent stronger bond strengths (HWI; Supplementary Table S6). Circle size is based on an individual’s normalized *bStrength* value (color figure online)

In the first two time periods, one trio in each second-order alliance had higher *bStrength_N* values than other males: indicating these trios played a greater role in maintaining the third-order alliance (these individuals have larger node size and are more central between alliances in Fig. 2a, b). All individuals in this social network participated in some third-order associations, although four individuals (REA, LAN, BAR, KRO) had particularly low *bStrength_N* values (Fig. 2a–c). Interestingly, these differences in *bStrength_N* values in the PD and KS alliances diminished in the third period, with the development of the KS–RR third-order alliance (Figs 2 and 3); and coincident with the loss of a central PD member.

### Temporal changes

#### T1: 2009–2010

During T1 (Fig. 2), the third-order alliance between the 7-member PD and 12-member KS alliances was already present (Q = 0.352 at HWI = 0.279; CCC = 0.966). RR males were not found in associations with PD or KS males during this period despite broadly overlapping ranges (Randić et al. 2012), and regular surveys (n = 14 RR surveys). Three males within the PD group, PRI, WAB, and NAT, showed the highest *bStrength* values, indicating their key role in maintaining the third-order alliance with KS (Fig. 2a; Supplementary Table S2). Within the KS alliance, MID, PON and QUA showed the highest *bStrength* values (Fig. 2a; Supplementary Table S2). The males in these two respective trios associated at high levels (HWI > 0.90; Supplementary Table S4) and were observed in multiple consortships, so were considered stable first-order alliances. The within-alliance relationships for all three second-order alliances are shown in Fig. 2d.

#### T2: 2011–2012

Two of the KS males (BOL and MID) were not sighted after the end of the 2010 field season and are presumed dead. The third-order alliance relationship between the now 10-member KS and 7-member PD alliance was maintained through T2 (Q = 0.462 at HWI = 0.213, CCC = 0.985; Fig. 2b). The same three PD individuals as in T1 showed high *bStrength* values (PRI WAB NAT). Within KS, two of the males with the highest *bStrength* values from T1 (PON QUA) were again part of the trio with the highest *bStrength* values, and PAS moved into the role previously occupied by MID (Fig. 2b). The PD male BAR had few sightings (n = 4) with KS and PD males. The within-alliance associations for all three second-order alliances over T2 are shown in Fig. 2e. RR males were not found in association with PD or KS males during this period (n = 35 RR surveys).

#### T3: 2013–2014

The most notable shift among the alliances happened between the T2 and T3 periods. The weakly-bonded PD male BAR and the central PD male PRI were observed through 2012 but disappeared prior to the start of the 2013 field season, leaving five PD males. From August through mid-November 2013, there were no surveys or follows that included RR males with KS or PD males other than in a foraging capacity, like the T1 and T2 periods (despite having recorded RR males in 29 surveys during the same period). In mid-November 2013 (aligning with the time of peak mating season), we observed a sudden inclusion of RR, with KS most frequently, though also with PD, in all behavioral contexts (Figs 2c and 3). These associations have continued through 2021 and 2022, the most recent field seasons (King et al. 2021 and unpublished data).

Modularity and hierarchical cluster analysis of this period assigned individuals to three clusters, corresponding to the KS, PD, and RR groups identified by observers (Q = 0.3112 at HWI = 0.1296, CCC = 0.9601). The new relationship was primarily between KS and RR. KS and PD were sighted together in surveys on 9 days, KS and RR on 11 days and PD and RR on only 2 days. Interesting shifts occurred in terms of *bStrength* centrality: the three RR males observed most with the KS and PD groups showed the highest overall, unnormalized *bStrength* values (Supplementary Table S2). During this period, we observed the RR alliance shift from a five-member group to a trio, with LAN at first associating and participating in consortships, but eventually SMO URC COO becoming the most observed RR trio. The fifth RR member, REA, was a much older male who had joined the RR group in 2010 after his previous second-order allies had disappeared and was presumed dead after the 2014 season.

Within the KS alliance, the same three individuals (PON QUA PAS) as in T2 showed the highest *bStrength* values within their alliance, but their relative prominence was diminished (Figs 2c and 3). Among PD males, NAT and WAB, former first-order allies of PRI, showed a notable decrease in *bStrength*, while the remaining three males in the PD alliance increased their *bStrength* relative to T2 (Figs 2b, c and 3). Within the PD alliance, additional shifts were apparent: overall *wStrength* declined for all PD males, as expected, due to the loss of PRI and BAR. More interestingly, the average HWI between the two PD first-order alliances (now a pair and a trio) declined precipitously from 0.717 in T2 to 0.132 in T3 (Fig. 2f).

### Behavioral interactions during third-order alliance fusions

#### Affiliative contact behavior

On 9 days during T3, we captured 10 fusion events between two first-order alliances from different second-order alliances using combined aerial and side-video (Table 1). The modal duration for our post-fusion analyses was 3 min; but we were able to observe one for nearly 10 min. Observations were terminated for a variety of reasons, including the groups separating or dispersing to forage, another fusion event occurring, or poor observation conditions. In 8 of the 10 fusion events, both first-order alliances were consorting a female, in the other two only one first-order alliance was consorting a female. Seven fusions were between KS and RR members, two were between KS and PD, and one fusion was between PD and RR. All 5 PD, 4 of 5 RR and 8 of 10 KS members were involved in the fusion events, seven of which included third-order petting or rubbing interactions (Table 1). 20 unique third-order petting/rubbing pairs were observed, including 13 KS–RR pairs, 6 KS–PD pairs and 1 PD–RR pair. One of these (KS–RR) involved a KS male that was not part of the fusion between the two first-order alliances, but who was in the area and appeared in the group during observations. During these same 10 events, we observed 13 intra-alliance petting or rubbing pairs (seven KS, four RR and two PD). The numbers of both third- and second-order alliance pairs is increased by including observations in which individuals were within touching distance, but actual contact could not be discerned (‘D0’; Table 1).

**Table 1:** The number of unique male pairs that engaged in petting or rubbing interactions (PR pairs) and synchronous surfacing (Synch pairs) during fusion events

Event	Alliances	Third-order	Third-order	Second-order	Second-order	Third-order	Second-order
		PR pairs	PR + D0 pairs	PR pairs	PR + D0 pairs	Synch pairs	Synch pairs
1	KS–RR	5	6	5	5	1	KS (1) RR (1)
2	KS–RR	0	0	0	1	0	0
3	KS–RR	0	1	0	1	0	RR (1)
4	KS–RR	5	7	5	5	0	0
5	KS–RR	0	0	0	2	0	KS (2)
6	KS–RR	1	2	0	2	1	0
7	KS–RR	1	1	2	3	0	0
8	PD–KS	2	4	1	2	0	KS (1)
9	PD–KS	4	6	2	3	1	KS (2) PD (1)
10	PD–RR	2	4	0	2	2	RR (1)

PR + D0 columns show the number of unique PR pairs when ‘D0’ behavior, where individuals were within touching distance, but actual contact could not be discerned, were included. The number of unique second-order synch pairs observed in each alliance is included in parentheses

#### Synchrony

During the same set of fusions, we scored 22 cases of individuals surfacing or diving side-by-side synchronously (‘synch’; Table 1), including eight cases of synchrony between third-order allies comprised of five unique pairs (two KS–RR, two PD–RR, one PD–KS). The other 14 cases of synchrony were between second-order allies and comprised 11 unique pairs (six KS, three RR, two PD; Table 1).

#### Interactions with females

We observed males from one alliance interact with the female being consorted by their third-order allies during 5 of the 10 fusions (three KS–RR, one PD–RR, one PD–KS). There were two petting or rubbing interactions: rubbing between a KS male and the female consorted by RR, and petting between an RR male and the female consorted by PD. First-order alliances often travel with consorted females in ‘formation’, a male trio side-by-side behind the female or a pair of males on either side and slightly behind (Connor et al. 1992a). During three fusions, we observed members of one alliance briefly in formation behind the other alliance’s female consort: KS with PD’s female consort, RR with PD’s female consort and, in the third fusion, KS and RR each swam in formation behind the other group’s consorted female.

## Discussion

We have demonstrated that third-order alliance relationships among male Indo-Pacific bottlenose dolphins in Shark Bay exhibit three key characteristics expected of ‘strategic’ alliances: they are differentiated, shift through time, and include affiliative interactions. Associations were not maintained equally by all males, but rather, certain first-order alliances played key roles in maintaining third-order alliances. Affiliative interactions did, nevertheless, occur broadly among third-order allies. We were fortunate to capture a third-order alliance forming when a trio from the RR alliance began associating with KS and PD; then primarily associated with KS. We discuss each of these phenomena below.

### Differentiated alliance relationships

The number and variety of differentiated relationships are considered important metrics of social complexity (Freeberg et al. 2012; Bergman and Beehner 2015; Fischer et al. 2017). The strength of associations between males in PD and KS in T1 and T2 were certainly differentiated but invite a simpler model. Based on *bStrength* alone, it is possible that third-order alliance bonds were maintained exclusively by a subset of individuals in each alliance. For example, the alliance between KS and PD in 2011–2012 (Fig. 2b) might have been based exclusively on bonds between the two relatively stable trios, with the other members ‘along for the ride,’ linked only through their second-order alliance bonds with these key individuals. However, this phenomenon was not apparent in T3 and our data on affiliative interactions did not support this model (below).

We have previously shown that relationships within first- and second-order alliances are differentiated; relatively stable trios often contain an ‘odd male out’ who is less frequently present, and when he is, less frequently synchronous with the other two males (Smolker et al. 1992; Connor et al. 2006). Males that spend more time together in second-order alliances engage in higher rates of affiliative contact behavior (Chereskin et al. 2022). Individuals exhibit marked preferences for first-order partners and individuals with more stable first-order associations have higher consortship rates (Connor et al. 2001; Connor and Krützen 2015). Overall, males with stronger and more homogenous bonds within their second-order alliance consort females more often and secure more paternities (Gerber et al. 2022; Connor et al. 2022). In Shark Bay, the alliance levels themselves, distinguished by association patterns and function, evince differentiated relationships. We previously considered the cognitive challenges for individuals negotiating a multi-level alliance system, where decisions at one level may impact success at others (Connor et al. 1992a; Connor 2007). Navigating three levels of *differentiated alliance relationships* should further increase the cognitive burden.

### Alliance shifts

If alliances and coalitions are strategic, we should see evidence that individuals occasionally form new alliance relationships, as male chimpanzees famously do when they compete for rank (de Waal 1982; Nishida 1983). We previously documented several types of alliance shifts in first- and second-order alliances. The first-order alliances of some males are stable, while others often change partners between consortships (Connor et al. 1992a, b, 2001). Connor et al. (1992b) documented a second-order alliance shift at the conclusion of a three-year competition among three stable first-order alliances. Individual additions and evictions from second-order alliances are uncommon but strategically important; for example, when an alliance of six or nine members that forms first-order trios only, loses and then replaces a member so all members can form trios again (Connor and Krützen 2015).

In this study, we documented a third-order alliance formation when RR began associating with KS. RR matured and were consorting females from 2002 but had shown no sign of a third-order association with KS until 2013. This change occurred in conjunction with the disappearance of two PD and KS members and, thus, may have helped mitigate declining second- and third-order alliance competitive ability. Two other members of RR did not join this new third-order alliance relationship (King et al. 2021); the older one died after 2014 and the other was no longer a member. Notably, the loss of PRI from the PD alliance was associated with the loss of the prominent inter-group position for PRI’s partners WAB and NAT and a weakening of their within-alliance bond with the other PD trio. One might have predicted that the diminished RR, KS and PD alliances would merge into one or two second-order alliances, but that has not happened; PD remained a second-order alliance and the KS–PD–RR triangle persisted through 2021 (King et al. 2021).

### Affiliative interactions

Affiliative interactions play a key role in social bond formation and maintenance, including coalitions and alliances within groups (Seyfarth and Cheney 2012). We have previously documented affiliative interactions within second-order alliances, both among members of the same and different first-order alliances (Connor et al. 2006; McCue et al. 2020; Chereskin et al. 2022). Our observations here of affiliative interactions during third-order group fusions contradict a model that simplifies third-order alliance relationships to a subset of individuals in each group. It is striking indeed that we recorded affiliative interactions in so many different third-order pairs (22 combining physical contact and synchrony) in such a small slice of the dolphins’ social lives (10 fusions totaling 39 min of observation). Males with among the lowest *bStrength* values for each alliance are found in the affiliative physical contact and synchrony pairs. This suggests that routine bond maintenance is an important and ongoing feature of third-order alliance behavior among the majority, if not all, members of each second-order alliance.

Previously, we found that males respond strongly to signature whistle playbacks of second-order allies irrespective of bond strength (King et al. 2021) and use whistle exchanges to maintain weaker second-order alliance bonds, while engaging in more affiliative contact among more strongly bonded second-order allies; thus alleviating the associated time and energy costs of petting by allowing males to ‘bond at a distance’ (Chereskin et al. 2022). These studies, combined with our finding that affiliative contact behaviors are widespread among third-order allies suggest the following generality: males maintain bonds with allies across three alliance levels that are strongly differentiated but universally valued.

The phenomenon of one male trio petting with and positioning itself in formation behind the consorted female of third-order allies is especially interesting, but difficult to interpret. The behavior could reflect dominance, except that it was reciprocated in one instance, or perhaps a form of sharing or ‘testing the bond’ (Zahavi 1977; Smuts and Watanabe 1990; Perry 2011). The few observations of aggression during fusion events, and the theft between third-order allies (ESM), are in keeping with the concept that alliances are predominantly cooperative but include conflicts. Previously we documented rare thefts within second-order alliances (Connor et al. 2011; Connor and Krützen 2015). It is notable that the single aggressive ‘tiff’ observed (ESM) was between second-order allies after joining third-order allies and the subsequent aggression was followed by an affiliative interaction.

The cognitive challenges of navigating three alliance levels are further demonstrated by the nature and pattern of social behaviors observed within these brief fusion events. The frequency and co-occurrence of these behaviors (affiliative contact, synchrony, behavior towards others’ consorts) among many different pairs indicates concurrent forms of ‘social grooming’ (Silk et al. 2003), social signaling (e.g., Bekoff 1995), and ‘bond testing’ (Zahavi 1977), within- and between-second-order alliances during fusion events; a period of potentially high uncertainty between third-order allies that are sometimes foes.

### Dolphins, bonobos and humans

Bonobos (*Pan paniscus*) present a striking exception to our characterization of interactions between primate social groups as typically hostile ‘us against them’ affairs. Bonobo groups have overlapping home ranges with variable and, thus, differentiated relationships among groups (Furuichi 2020; Samuni et al. 2022). Encounters often include affiliative sexual behavior and even coalitions between individuals of different groups (Tokuyama et al. 2019; Furuichi 2020). However, inter-group bonobo coalitions were generally two or a few from both groups against another bonobo from one of the same two groups (Tokuyama et al. 2019), rather than one or two groups against another group, as occurs in the dolphin second- and third-order alliance interactions (Connor et al. 2011). The three levels of strategic alliances among unrelated individuals in dolphins is unique outside humans, although the route to the evolution of ‘alliances of alliances’ in humans and dolphins likely differed (Connor et al. 2022). The evolution of between group alliances in humans is thought to have depended on the presence of kin and affines in allied groups (Chapais 2013); in the Shark Bay dolphins, affiliative interactions among non-relatives may be sufficient to establish and maintain inter-alliance bonds (Samuni et al. 2022; Connor et al. 2022).

## Conclusion

The occurrence of three levels of strategic alliances among unrelated individuals is unique outside of humans. Here, we demonstrated that male dolphins in Shark Bay maintain third-order alliance relationships that are differentiated, shift through time, and are mediated by affiliative interactions. These characteristics are typical of intra-group coalitions between individual male primates, like chimpanzees, that use coalitions to compete for rank, and thus increase reproductive success (Wroblewski et al. 2009; Gilby et al. 2013). In the dolphins, alliance behavior is associated with striking differences within and between second-order alliances in the rate that males consort females and in bond strength (Connor et al. 2001, 2017; Connor and Krützen 2015), which predicts reproductive success (Gerber et al. 2022). With this study, we can now state unequivocally that the same complexities of alliance and coalition formation that are present in one alliance or coalition level within some other mammalian social groups, are present in all three levels of male dolphin alliance formation. Alliances that Chapais (1995) said may generate “the most complex social structures in the world” have, outside of humans, reached a pinnacle in Shark Bay’s Indo-Pacific bottlenose dolphins.

## Supplementary Information

Below is the link to the electronic supplementary material.

Supplementary file1 (DOCX 2321 KB)

## Data Availability

Materials used in this analysis can be found at https://github.com/whitneyf/friedman_etal_2023_anicog.
