# Supplementary material for: Inter-group alliance dynamics in Indo-Pacific bottlenose dolphins (Tursiops aduncus)
Source: Anim Cogn. 2023 Jun 30;26(5):1601–12. doi: 10.1007/s10071-023-01804-y (PMC10442264; doi:10.1007/s10071-023-01804-y)
Supplement: Supplementary file 1 — Supplementary file1 (DOCX 2321 KB) [file 10071_2023_1804_MOESM1_ESM.docx]

**Supplementary Material**

*Corresponding manuscript*

W. R. Friedman, M. Krutzen, S. King, S. Allen, L. Gerber, S. Wittwer, and R. C. Connor. (2023) Inter-group alliance dynamics in Indo-Pacific bottlenose dolphins (*Tursiops aduncus* ). *Animal Cognition.*

*Corresponding authors*

Whitney R. Friedman <wrfriedm@ucsc.edu>, Richard C. Connor < rconnor@umassd.edu>

***Data Collection***

The use of Unoccupied Aerial Vehicles (UAVs), or drones, in wildlife research has become so prevalent in recent years (e.g. Barnas et al. 2020; Christie et al. 2016; Hodgson et al. 2017) that readers may be unfamiliar with the relative difficulty of obtaining aerial video with earlier methods (e.g. Hodgson 2010; Nowacek et al. 2001). Coordinating aerial and side-video with the tethered helikite was challenging as it required maneuvering the boat with respect to the wind, sea and dolphins such that the dolphins remained within the field of view of the fixed helikite-mounted camera while the boat was alongside the dolphins for individual dorsal fin identification. The fine-scale behavioral interactions we quantified with this method proved invaluable here. Our research now involves the routine use of UAVs to supplement boat-based observations, or indeed to conduct playback experiments combining acoustic recordings, boat-based observation and aerial recording (e.g. Allen et al. 2017; King et al. 2021, respectively).

***Aggression and socio-sexual behavior***

Although the focus here is on the occurrence of affiliative interactions between third-order allies, it is important to consider the context of affiliative interactions with respect to instances of aggression and socio-sexual behavior (which may range from affiliative to aggressive, Connor et al., 2000). Socio-sexual behavior tends to be more vigorous and includes mounting, ‘goosing’ (one individual probes its rostrum into the genital area of another) and ‘push ups’, where one male is pushed partly up out of the water by one or more individuals. During socio-sexual encounters, males more commonly goose females and mount males (Furuichi et al., 2014). Aggression includes chasing, biting, hitting, tiffs (head-to-head posturing with head shaking that may escalate into fighting) and fighting (rapid, multiple attempts to hit and bite and avoid the same). The more vigorous movement associated with socio-sexual behavior and aggression produces more splashing, rendering the identification of participants more difficult.

Aggression and socio-sexual behavior, along with aggressive vocalizations, were documented during one PD-RR fusion in a sequence that also included affiliative behavior. During the first 10 s of the fusion, there was a tiff between two PD members. Then, a PD male (WAB), with an RR male alongside, approached another RR male (URC), biting his peduncle then chasing him. Within 60 s, the same two males (WAB, URC) were petting, then they synched (performed synchronous movements) together while there was a mount involving another PD-RR pair. More intense social behavior followed but we were unable to identify specific individuals. During the course of this study we observed several third-order interactions where KS, PD and RR males were ‘on the same side’, as well as an interesting conflict between these second-order alliances. While two PD males (NAT and WAB) were consorting a female, the RR trio joined and initiated intense social behavior. Eight mins later, the other PD trio joined, and a further three mins later, six KS males. At 33 mins after the initial PD-RR fusion, one KS trio left with the female originally consorted by the PD males (NAT and WAB). These conflicts between third-order allies exemplify the dynamic nature of third-order relationships (see also Connor et al. 2011).

***Resight Definition:*** A ‘resight’ is the repeated survey of a group of animals with identical membership, within 2 hours of surveying the same group

**Table S1**: Estimated ages as of 01-Nov-2009 for the 24 males within three second-order focal alliances (KS, PD, RR).

| **ID** | **Alliance** | **Estimate age** |
| --- | --- | --- |
| BOL | KS | 25.0 |
| CEB | KS | 23.0 |
| DEE | KS | 23.0 |
| DNG | KS | 24.0 |
| IMP | KS | 27.0 |
| KRO | KS | 30.0 |
| MID | KS | 20.0 |
| MOG | KS | 27.0 |
| NOG | KS | 29.0 |
| PAS | KS | 24.0 |
| PON | KS | 28.0 |
| QUA | KS | 21.1 |
| BAR | PD | 37.0 |
| BIG | PD | 34.0 |
| FRE | PD | 34.0 |
| NAT | PD | 29.0 |
| PRI | PD | 30.0 |
| RID | PD | 21.0 |
| WAB | PD | 31.0 |
| COO | RR | 21.1 |
| LAN | RR | 20.0 |
| REA | RR | 37.0 |
| SMO | RR | 20.9 |
| URC | RR | 19.1 |

**Table S2:** The between alliance strength (*bStrength*) values and within-alliance strength (*wStrength*) values for the three second-order alliances (KS, PD, RR) over two-year intervals (2009-2010, 2011-2012 and 2013-2014).

|  |  | bStrength | | | wStrength | | |
| --- | --- | --- | --- | --- | --- | --- | --- |
| ID | Alliance | 0910 | 1112 | 1314 | 0910 | 1112 | 1314 |
| BOL | KS | 0.91 | - | - | 5.3 | - | - |
| CEB | KS | 0.85 | 0.75 | 1.36 | 5.28 | 5.88 | 4.6 |
| DEE | KS | 0.92 | 0.57 | 1.42 | 5.17 | 5.7 | 4.81 |
| DNG | KS | 1.12 | 0.54 | 0.81 | 4.35 | 5.97 | 3.57 |
| IMP | KS | 0.58 | 0.81 | 1.19 | 4.74 | 5.9 | 4.6 |
| KRO | KS | 0.99 | 0.58 | 0 | 4.12 | 5.28 | 0.25 |
| MID | KS | 1.63 | - | - | 4.82 | - | - |
| MOG | KS | 0.83 | 0.85 | 1.13 | 4.75 | 6 | 4.42 |
| NOG | KS | 1.33 | 0.52 | 0.67 | 4.14 | 5.62 | 2.72 |
| PAS | KS | 1.2 | 1.5 | 1.69 | 5.08 | 4.87 | 4.59 |
| PON | KS | 1.71 | 1.35 | 1.45 | 5.6 | 5.38 | 4.84 |
| QUA | KS | 1.8 | 1.35 | 1.52 | 5.55 | 5.38 | 4.8 |
| BAR | PD | 0.64 | 0.54 | - | 2.22 | 1.28 | - |
| BIG | PD | 1.49 | 1.02 | 1.39 | 3.86 | 4.48 | 1.79 |
| FRE | PD | 1.41 | 1.02 | 1.25 | 3.84 | 4.48 | 1.82 |
| NAT | PD | 2.93 | 1.72 | 1.09 | 3.89 | 4.24 | 1.29 |
| PRI | PD | 3.06 | 1.72 | - | 3.79 | 4.24 | - |
| RID | PD | 1.41 | 1.08 | 1.37 | 3.81 | 4.34 | 1.98 |
| WAB | PD | 2.93 | 1.72 | 1.12 | 3.89 | 4.24 | 1.3 |
| COO | RR | 0 | 0 | 1.83 | 2.15 | 3.05 | 2.47 |
| LAN | RR | 0 | 0 | 0.27 | 2.66 | 2.58 | 1.93 |
| REA | RR | 0 | 0 | 0.24 | 0.15 | 2.03 | 0.96 |
| SMO | RR | 0 | 0 | 1.84 | 2.6 | 2.95 | 2.49 |
| URC | RR | 0 | 0 | 1.96 | 2.6 | 2.99 | 2.55 |

Table S3. Association values (HWI) between KS, PD, and RR males from 2009-2014


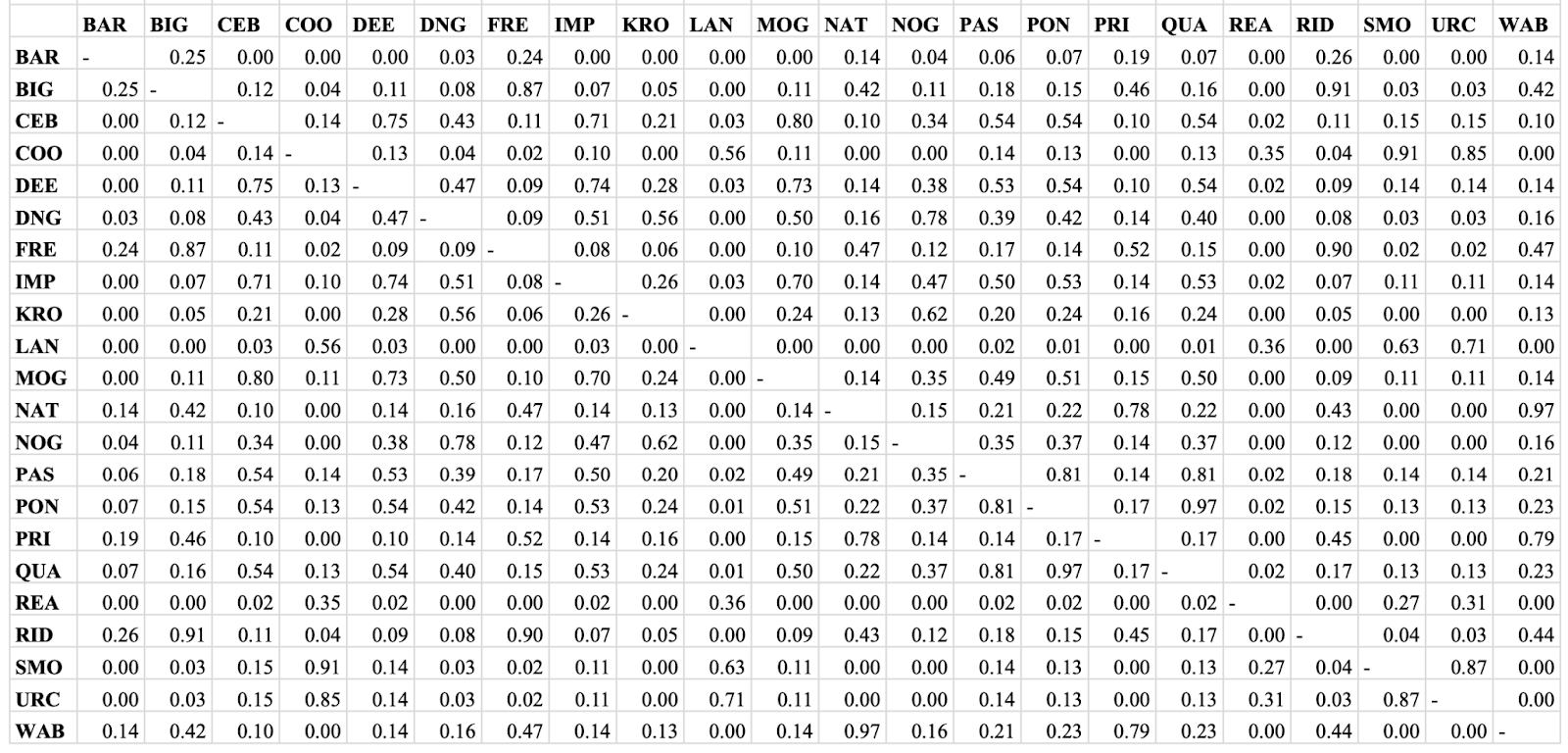


Table S4. Association values (HWI) between KS, PD, and RR males from 2009-2010


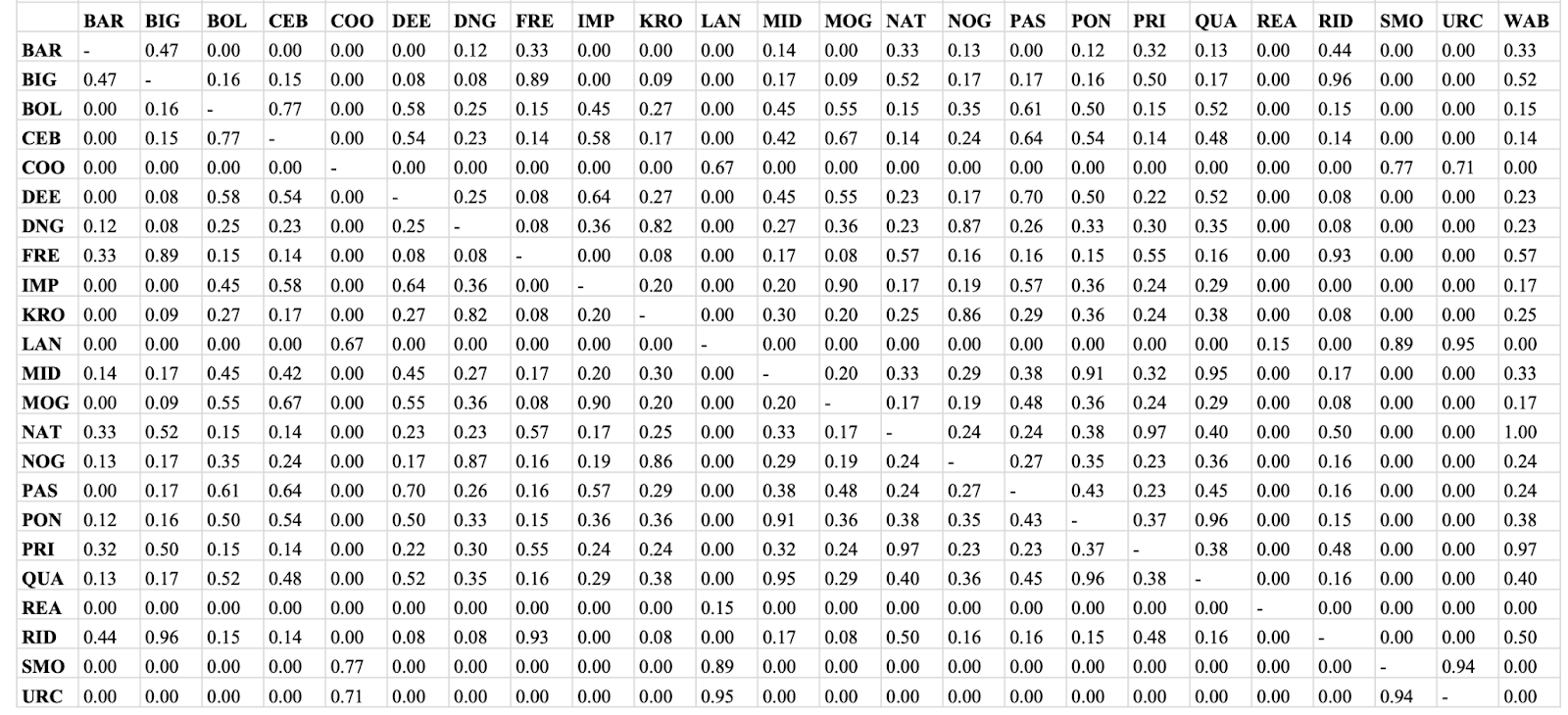


Table S5. Association values (HWI) between KS, PD, and RR males from 2011-2012


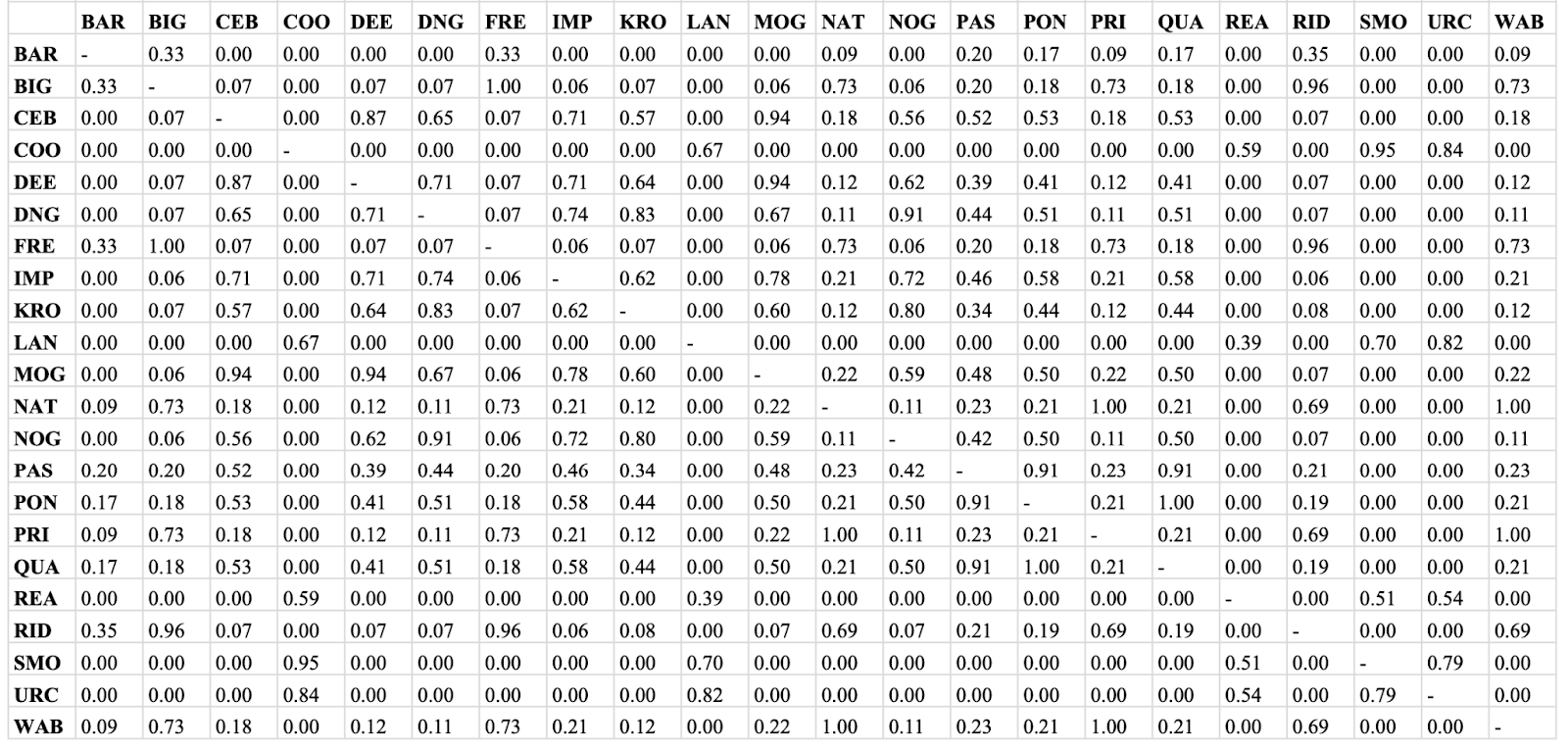


Table S6. Association values (HWI) between KS, PD, and RR males from 2013-2014


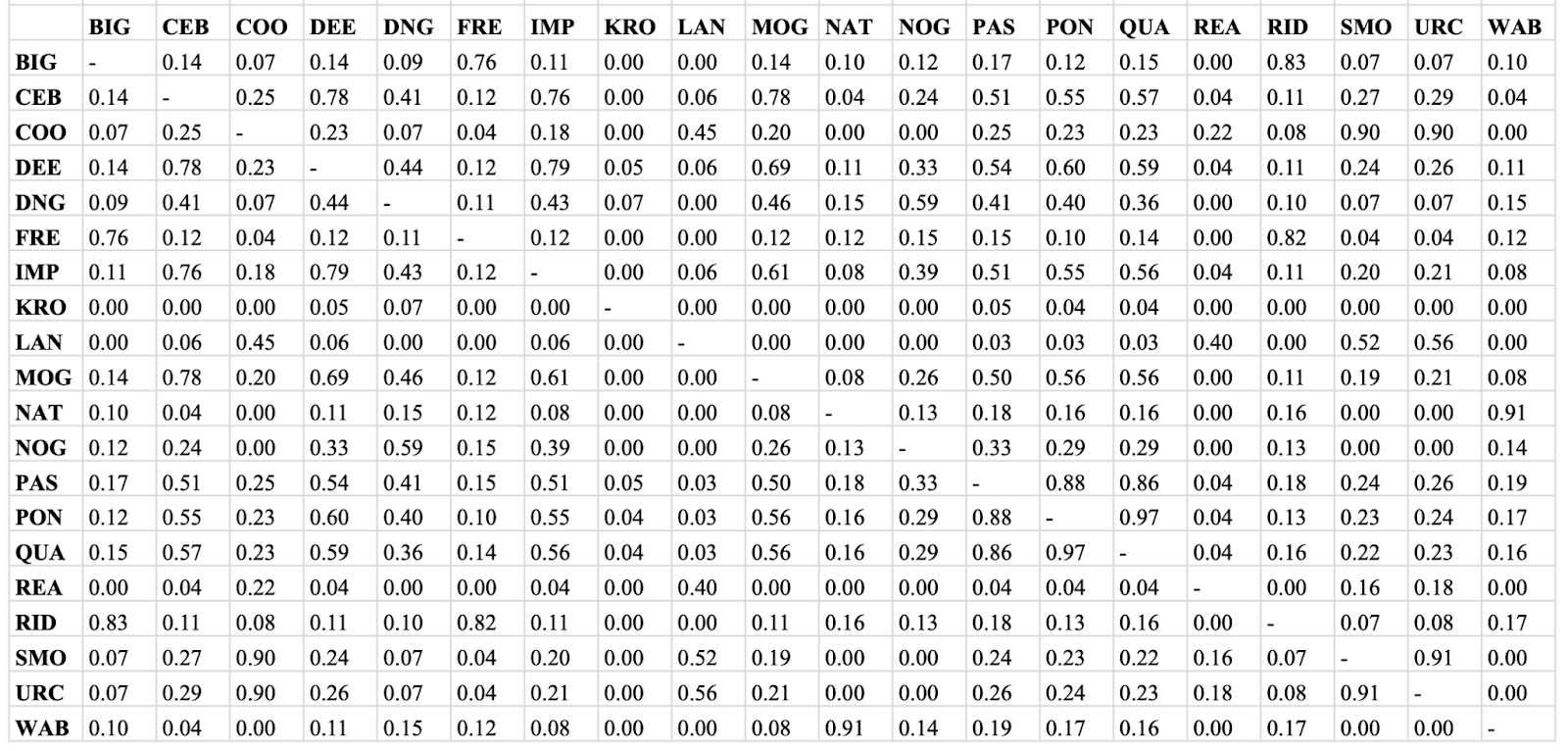


**References**

Allen SJ, King SL, Krützen M, Brown AM 2017. Multi-modal sexual displays in Australian humpback dolphins. *Scientific Reports* 7: 13644.

Barnas, A.F., Chabot, D., Hodgson, A.J., Johnston, D.W., Bird, D.M. and Ellis-Felege, S.N. (2020). A standardized protocol for reporting methods when using drones for wildlife research. *Journal of Unmanned Vehicle Systems* 8(2): 89–98.

Christie, K.S., Gilbert, S.L., Brown, C.L., Hatfield, M. and Hanson, L. (2016). Unmanned aircraft systems in wildlife research: Current and future applications of a transformative technology. *Frontiers in Ecology and the Environment* 14(5): 241–251.

Connor, R. C., Wells, R. W., Mann, J., & Read, A. J. (2000). The Bottlenose dolphin: Social relationships in a fission-fusion society. In J. Mann, R. C. Connor, P. L. Tyack, & H. Whitehead (Eds.), *Cetacean Societies* (pp. 91–126). Chicago, IL: The University of Chicago Press.

Connor, R. C., Watson-Capps, J. J., Sherwin, W. B., and Krützen, M. (2011). A new level of complexity in the male alliance networks of indian ocean bottlenose dolphins *(Tursiops sp.)*. *Biol Lett*, 7(4):623–626.

Furuichi, T; Connor, R & Hashimoto, C. 2014. Non-conceptive sexual interactions in

Monkeys, Apes and Dolphins. In (J. Yamagiwa & L. Karczmarski, eds.); *Primates*

*& Cetaceans: field research and conservation of complex mammalian societies*. pp.

385-408. Tokyo: Springer.

Hodgson, A. (2007). “Blimp-cam”: Aerial video observations of marine animals. *Marine Technology Society Journal* 41(2): 39-43.

Hodgson, A., Peel, D. and Kelly, N. (2017). Unmanned aerial vehicles for surveying marine fauna: Assessing detection probability. *Ecological Applications* 27(4): 1253–1267.

King, S.L., Connor, R.C., Krützen, M. Allen, S. (2021)*.* Cooperation-based concept formation in male bottlenose dolphins. *Nat Commun* **12,**2373

Nowacek, D. P., Tyack, P. L. and Wells, R. S. (2001). A platform for continuous behavioral and acoustic observation of free-ranging marine mammals: overhead video combined with underwater audio. *Mar Mammal Sci* 17(1): 191-199.

Connor RC, Smolker RA, Richards AF. (1992) Two levels of alliance formation among male bottlenose dolphins (*Tursiops* sp.). *Proc. Natl. Acad. Sci. U. S. A.* **89**, 987–990.
